# Musculoskeletal Injuries and Outcomes Pre- and Post- Emergency Medicine Training Program

**DOI:** 10.5811/westjem.2019.7.41448

**Published:** 2019-10-14

**Authors:** Peter Mattson, Ezechiel Nteziryayo, Adam R. Aluisio, Michael Henry, Noah Rosenberg, Zeta A. Mutabazi, Jeanne D’Arc Nyinawankusi, Jean Claude Byiringiro, Adam C. Levine, Naz Karim

**Affiliations:** * Warren Alpert Medical School, Department of Emergency Medicine, Providence, Rhode Island; † Centre Hospitalier Universitaire de Kigali, Kigali, Rwanda; ‡ Columbia University Vagelos College of Physicians and Surgeons, New York, New York

## Abstract

**Introduction:**

Musculoskeletal injuries (MSI) comprise a large portion of the trauma burden in low- and middle-income countries (LMIC). Rwanda recently launched its first emergency medicine training program (EMTP) at the University Teaching Hospital-Kigali (UTH-K), which may help to treat such injuries; yet no current epidemiological data is available on MSI in Rwanda.

**Methods:**

We conducted this pre-post study during two data collection periods at the UTH-K from November 2012 to July 2016. Data collection for MSI is limited and thus is specific to fractures. We included all patients with open, closed, or mixed fractures, hereafter referred to as MSI. Gathered information included demographics and outcomes including death, traumatic complications, and length of hospital stay, before and after the implementation of the EMTP.

**Results:**

We collected data from 3609 patients. Of those records, 691 patients were treated for fractures, and 674 of them had sufficient EMTP data measured for inclusion in the analysis of results (279 from pre-EMTP and 375 from post-EMTP). Patient demographics demonstrate that a majority of MSI cases are male (71.6% male vs 28.4% female) and young (64.3% below 35 years of age). Among mechanisms of injury, major causes included road traffic accidents (48.1%), falls (34.2%), and assault (6.0%). There was also an observed association between EMTP and trends of the three primary outcomes: a reduction of death in the emergency department (ED) from those with MSI by 89.9%, from 2.51% to 0.25% (p = 0.0077); a reduction in traumatic complications for MSI patients by 71.7%, from 3.58% to 1.01% (p = 0.0211); and a reduction in duration of stay in the ED among those with MSI by 52.7% or 2.81 days on average, from 5.33 to 2.52 days (p = 0.0437).

**Conclusion:**

This study reveals the current epidemiology of MSI morbidity and mortality for a major Rwandan teaching hospital and the potential impacts of EM training implementation among those with MSI. Residency training programs such as EMTP appear capable of reducing mortality, complications, and ED length of stay among those with MSI caused by fractures. Such findings underscore the efficacy and importance of investments in educating the next generation of health professionals to combat prevalent MSI within their communities.

## INTRODUCTION

Musculoskeletal injuries (MSI) are a major cause of morbidity and mortality across the world that disproportionately affect those in low- and middle-income countries (LMIC), which often lack trained healthcare providers who can properly treat such conditions.^1^,^2^ Approximately 90% of the five million annual deaths across the world due to injuries occur in LMICs such as Rwanda.^3^,^4^ The literature lacks an updated fund of knowledge regarding the prevalence, etiology, and treatment for MSIs in Rwanda to supplement previous studies. The growing number of Rwandan healthcare providers may incorporate this knowledge into educational programs when approaching MSI.^1^,^2^,^3^

Injuries in Rwanda are associated with significant morbidity and mortality.^5^,^6^ Past studies in Rwanda have shown that most trauma victims are young men.^5^,^6^ Road traffic accidents (RTA), especially those involving motorcycles, were the most common mechanism for adults, while children were frequently injured as pedestrians.^3^ Approximately one-quarter of injured patients suffered a fracture.^3^ The overall mortality prevalence was 5.5% with approximately half of the hospital deaths occurring in the emergency department (ED).^7^ Yet, these mortality figures do not paint a comprehensive picture of the burdens posed by MSIs and fractures in particular.

MSIs resulting from trauma are frequently undertreated, causing difficulty for patients to resume normal work and life activities.^8^ This is related both to cost and a shortage of technology and supplies.^9^ In addition to a dearth of supplies, achieving health outcome targets without securing the appropriate human resources is difficult.^10^,^11^ One team in Namibia found that three out of the eight Millennium Development Goals concerning healthcare required appropriate human resources for success.^10^ A recent interrupted time-series study found that building Rwanda’s emergency medicine training program (EMTP) resulted in an absolute reduction of overall facilities-based mortality by 4% overall, which was twice as great a decline as the national trend.^12^ Such investments are vital to improving health in this region. While Africa contains approximately one-quarter of the world’s burden of diseases, it possesses 4% of its health staff.^11^,^13^

A recent systematic review found that of 59 LMIC emergency care programs, very few incorporated specialist emergency care training. 14 The largest share of facilities was staffed either by physicians-in-training or by physicians whose level of training was unspecified. Data showed high patient loads and mortality, specifically in Africa where a substantial proportion of total deaths occurred in EDs. Compared to other LMIC regions, ED mortality is highest in Africa, with a median mortality rate of 3.4% compared to the average of 1.8% across all studied LMICs.^14^,^15^ A minority of LMIC EDs incorporate specialty-trained emergency physicians into the staffing paradigm, but availability is limited.^14^ The high volume and urgency of treatment make emergency care an important area of focus for interventions aimed at reducing mortality in these settings.

Within a short period of time, Rwanda has made significant improvements to its healthcare system. Rwanda’s transformation of its health sector since the 1990s has helped to raise life expectancy from 27 years to 63 years of age, and nearly all Rwandans have health insurance.^16^,^17^ Although there have been significant improvements, Rwanda has just 0.84 health providers per 1000 population, the majority of whom are generalists. This number falls below the minimum 2.3 providers per 1000 population set forth by the World Health Organization.^13^. In 2011, the Rwandan Ministry of Health began a seven-year partnership with a U.S. academic consortium to train Rwandan providers to become future educators through medical residencies, creating the Human Resources for Health (HRH) Program.

**Population Health Research Capsule**

What do we already know about this issue?

*Past studies in Rwanda have detailed the epidemiology of musculoskeletal injuries (MSI), such as the demographics and etiology of these cases*.

What was the research question?

What is the effect of an emergency medicine training program (EMTP) on mortality, complications, and emergency department (ED) length of stay among those with MSI?

What was the major finding of the study?

*The EMTP in Rwanda reduced mortality, complications, and ED length of stay among those with MSI caused by fractures*.

How does this improve population health?

*Determining MSI epidemiology and effects of an EMTP on a major health burden in Rwanda demonstrates one approach to improve health outcomes on a population level*.

Among the new medical residencies is the first EMTP in Rwanda.^16^ These trainees have introduced new emergency skills, such as triage and resuscitation, along with improvements to local protocols and systems.^12^ The training curriculum was in line with the American Board of Emergency Medicine (East Lansing, Michigan) 2013 Model of the Clinical Practice of Emergency Medicine.^17^ International faculty practicing EM were hired to implement EM training through the HRH program, a collaboration between academic medical centers in the U.S. and the Rwandan Ministry of Health (MOH).^12^ Within the EMTP curriculum, specific longitudinal educational trainings on the diagnosis and treatments of MSI and fractures were provided through lectures and workshops.

Research studies regarding the epidemiology of injuries and the impact of emergency training on patient outcomes have been conducted, although specific epidemiology regarding fractures and the impact of training on patient outcomes is lacking.^14^,^16^ The purpose of our research was twofold: 1) to understand the epidemiology of MSI fractures in Rwanda; and 2) to evaluate the progress of the country’s first EM residency program in treating MSI-related injuries by assessing ED mortality rates, length of stay, and complication rates.

## METHODS

### Study design and setting

This was a pre-post study examining the characteristics and outcomes of MSIs before and after implementation of an EMTP at the University Teaching Hospital of Kigali (UTH-K) in Kigali, Rwanda. UTH-K is an urban referral and tertiary-care teaching hospital with approximately 560 inpatient beds and 40 ED beds. UTH-K contains a 24-hour Accident and Emergency Department (A&E) that serves adult patients with acute complaints, as well as pediatric and obstetric trauma patients. Resources at UTH-K include 24-hour surgical coverage, 24-hour access to radiologic services including radiograph, ultrasound, and computed tomography, as well as continuous access to general surgery, orthopedic and neurological specialists.^7^,^18^

The A&E department is covered by general practice physicians (GP) and EM residents. An EM post-graduate diploma program was initiated on November 1, 2013, and most physicians enrolled subsequently participated in the official EM residency, which began in September 2015. Both programs are herein formally referred to as the EMTP. Prior to initiation of these training programs, care was provided exclusively by GPs. Since initiation of EMTP, ED care has been provided jointly by GPs and EM resident-trainees who have oversight by board-certified emergency physicians.

All patients who presented at UTH-K during the two data collection periods, from November 2012–October 2013 and August 2015–July 2016, were eligible for inclusion. These pre- and post-time periods for data collection were chosen to correspond with the absence of an EMTP and implementation of an EMTP, respectively. We identified cases and queried data from institutional records via protocolled methods, as previously described in prior studies.^7^,^18^,^19^,^20^ Briefly, using a multipoint composite index generated from an electronic hospital database, we identified all cases during each month of the accruement periods. Subsequently, all cases were coded with a unique identification number and were sampled at random until a sufficient number of records meeting inclusion criteria were identified (range: 135–165 records per month). We then narrowed the dataset to those with MSI, either with open, closed, or mixed fractures. Next, we applied the following exclusion criteria: incomplete or erroneous evaluation documentation dates from the ED, comprising patients without admission dates, or patients with admission dates that preceded discharge dates.

Measured variables included age, sex, mechanism of injury, injury type, hospital vital signs, hospital admissions, surgical interventions, medical treatments, discharge date, and disposition. If more than one anatomical region was indicated as injured, each region was recorded.^8^ We did not collect post-discharge outcomes, such as subsequent emergency visits, hospitalizations, or post-discharge death,.

### Ethical consideration

The research study was approved by the College of Medicine and Health Sciences University of Rwanda Institutional Review Board (IRB) No 310/CMHS IRB/2017 and Rhode Island Hospital (Lifespan) IRB (4144114; 45 CFR 46.110.5).

### Data management

Data were initially collected from medical records and then abstracted and entered into REDCap, a standardized, secured, web-based data collection instrument.^21^ The database inputs were recorded by trained study personnel at UTH-K, verified by a trained physician, and then validated for any errors in order to meet inclusion and exclusion criteria.

### Data analysis

We performed analyses using Stata Statistical Software 14.2 (StataCorp, College Station, Texas). Continuous variables were summarized using medians with interquartile ranges or means with corresponding 95% confidence intervals. Categorical variables were reported as percentages using frequencies. Using two-sample t-tests with equal variance for patients treated before vs after implementation of EMTP, we compared outcomes for pre- and post-EMTP. Data were collected on information from the initial ED encounter and any subsequent hospitalization during the same stay.

## RESULTS

### Epidemiology

General MSI epidemiological findings in our 691 patients are outlined in supplements. A total of 17 patients were excluded for incomplete documentation. Of these records, 279 occurred before the start of the EMTP on November 1, 2013, while 395 occurred on or after the start of the program. Thus, patients were divided into pre-EMTP and post-EMTP groups resulting in 674 available patient records (see supplements). Patient demographics demonstrate that a majority of MSI cases were male (71.6%) and younger than 35 years of age (64.3%). Major mechanisms of trauma included RTAs (48.1%), falls (34.2%), and assault (6.0%). Of those involved in RTAs, a substantial proportion involved motorcycles (43.2%) while over one-quarter of accidents involved a pedestrian being struck (28.6%). The majority of patients were transported from another health facility (64.3%), while other patients were transported from the street (23.6%) or from home (9.0%).

Clinical characteristics of this cohort in Table 1 demonstrate approximately equal numbers of open and closed fractures (35.2% and 35.7%, respectively). The most common anatomical regions of these fractures and injuries included the lower extremity (52.7%), upper extremity (30.0%), craniofacial (6.8%), abdomen-pelvis (4.2%), and thorax (3.9%). The most common abnormal vital signs included tachycardia (22.1%), hypotension (6.0%), and tachypnea (4.7%). Approximately 1 in 10 patients had a Glasgow Coma Scale (GCS) score of 12 or below, with 24 patients’ scores ranging from 9–12 (5.2%) and 14 patient scores ranging from 3–8 (3.0%).

Care delivery metrics divided between ED outcomes and in-hospital outcomes are shown in Table 2. In the ED, a trauma intervention was performed for approximately three out of every four patients (76.0%). Most common trauma interventions included traction or splinting (52.2%), wound care (21.9%), and hemorrhage control (13.6%). Antibiotics and tetanus antitoxin were also commonly administered for fractures (84.4% and 26.4%, respectively), although they were more frequently given in the case of open fractures (86.6% and 54.3%, respectively).

Other common emergency procedures included analgesic medication (65.7%), intravenous liquid infusion (34.1%), and endotracheal intubation (28.1%), along with less common interventions such as transfusion of blood products (16.6%) and oxygen supplementation (9.2%). In over four out of every five cases, an emergency consultation was obtained (83.8%), most commonly from orthopedics (67.9%), acute care surgery (20.6%), and neurosurgery (10.0%). In a majority of cases, laboratory tests and imaging tests were ordered (62.8% and 82.5%, respectively). Nearly three of every four patients were admitted to the hospital (73.3%), with the most common admitting wards comprising orthopedics (65.6%), surgical (27.7%), and neurosurgery (5.1%).

As seen in Table 2, analysis of in-hospital care and outcomes showed that a majority of patients required operative management (88.1%). Most common procedures included open reduction (42.3%), wound debridement (22.7%), and closed reduction with external fixation (22.2%). A lesser percentage of the in-hospital patients required intensive care after admission (4.2%) or suffered from hospital complications (2.5%). Patient outcomes varied from discharges (89.6%) to transfers (7.3%) to deaths in hospital (2.4%).

### Impact of Emergency Medicine Training Program (EMTP)

Baseline characteristics in Table 3 highlight the similarities and differences among the total of 674 patients seen prior to and following the implementation of the EMTP. Several patient characteristics did not differ between the pre-EMTP and post-EMTP cohorts, including age, gender, proportion of open fractures, proportion of RTAs, heart rate, and systolic blood pressure.

**Figure 1:**
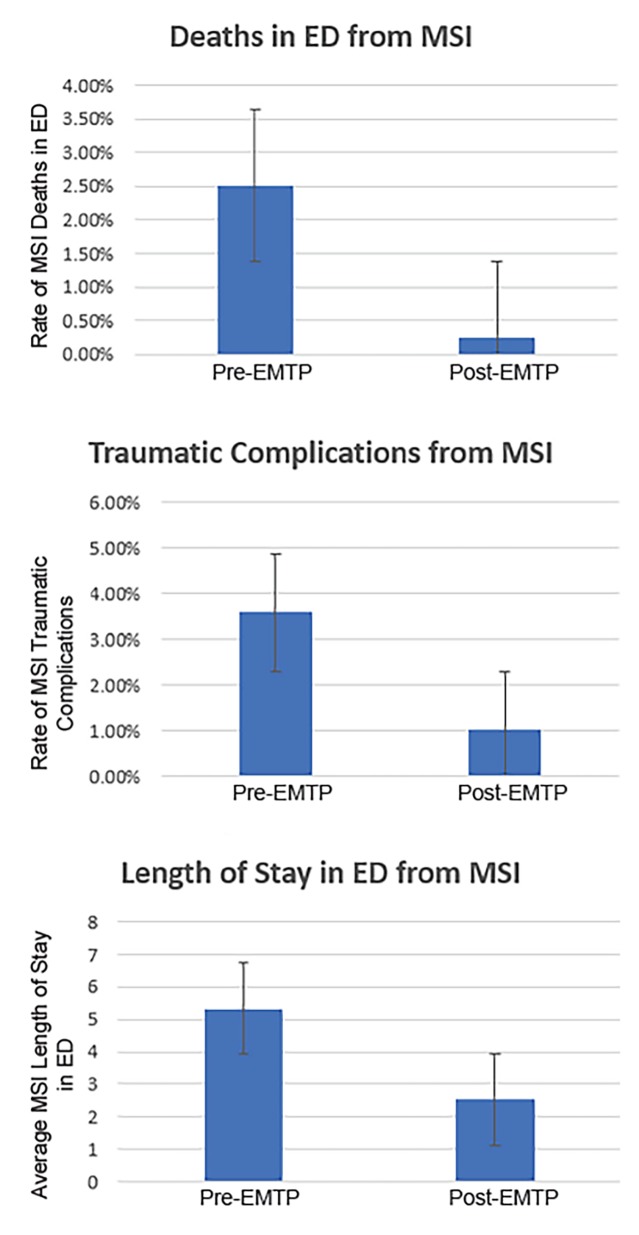
EM residency outcomes among MSI patients with musculoskeletal injuries show decreased death rate, complication rate, and duration of stay in emergency department. Using two-sample t-tests with equal variance for patients treated before vs after implementation of EMTP, results demonstrate a decrease and thus improvement in the three primary outcomes of interest. EMTP reduced the death rate in the ED from MSI by 89.9%, from 2.51% to .253% (p=0.0077). The program dropped traumatic complications from MSI by 71.7%, from 3.58% to 1.01% (p = .0211). Lastly, EMTP reduced duration of stay in the ED by 52.7% or 2.81 days on average, from 5.33 to 2.52 days (p = .0437). *Group 0 refers to pre-EMTP patients while group 1 refers to patients admitted during the EMTP program. *MSI*, musculoskeletal; *ED*, emergency department; *EMTP*, emergency medicine training program.

Overall, there was significant improvement in ED outcomes after the implementation of EM training at UTH-K. Results demonstrate improvement in the three outcomes of interest. Specifically, there was a decrease in the ED mortality prevalence in patients with MSI by 89.9%, from 2.51% to 0.25% (p = 0.0077). There was also a decrease in traumatic complications including wound infection, compartment syndrome, and associated shock from MSI by 71.7%, from 3.58% to 1.01% (p = 0.0211). Lastly, there was a reduction in the duration of stay in the ED by 52.7% or 2.81 days on average, from 5.33 to 2.52 days (p=0.0437) (Figure 1 and Table 4).

Similar measures, such as deaths and length of stay, did not significantly change in the in-hospital setting. There was a non-significant increase in the in-hospital mortality rate from 1.4% to 1.8% (p = 0.7331) and a non-significant decrease in in-hospital length of stay from 19.9 to 16.3 days (p = 0.0529) (Table 4). Several secondary outcomes in the ED setting also increased significantly, such as imaging requests (from 74.2% to 88.4%; p = 0.0000), laboratory exam requests (from 55.6% to 69.0%; p = 0.0003), and administered tetanus antitoxin (from 15.1% to 34.3%; p = 0.0000). Additionally, ED exams were recorded more often, such as the GCS (from 62.9% to 74.7%; p = 0.0012), medical history (from 52.3% to 62.0%; p = 0.0120), and physical exam (from 98.2% to 99.7%; p = 0.0362).

## DISCUSSION

In the population of patients seeking emergency care for MSI, this study found significant improvements in mortality and complication rates, length of stay, and an array of secondary outcomes in association with the implementation of EMTP. The training curriculum taught by EM faculty is thought to have played a key role in the improvement of these outcomes. This curriculum included specific longitudinal educational trainings on the diagnosis and treatments of MSI provided through lectures and workshops that all residents completed. These findings help to demonstrate the potential importance of investing in the training of formal EM specialists to address the large burden of morbidity and mortality associated with MSI in LMICs.

It has been previously proposed that relatively simple interventions in areas such as emergency triage, communication, and education and supervision could lead to reductions in LMIC mortality in the ED, where up to 10–15% of all deaths occur.^14^ The study demonstrates a temporal association between MSI outcomes in the ED and the inception of an EMTP, underlining the importance of developing such programs. While many LMIC governments do not list EM in their medical education priorities, they could consider doing so to tackle the treatment of such a high volume of patients with acute health problems.

The epidemiological results provide the first available data on MSI from a Rwandan hospital. Understanding the patient population, anatomical distribution of fractures, and mechanisms of injury could allow for more practical incorporation into the EMTP’s future MSI curriculum. This understanding may also aid in proper diagnosis and treatment of the growing burden of MSI cases, a critical step for improving patient outcomes. Moreover, these epidemiological results, to an extent, confirm those of another research team that studied traumatic injuries in Rwanda’s pre-hospital service, an epidemiological profile that showed nearly one-fourth (24%) of injured patients suffered from a fracture.^3^

Most importantly, the epidemiological patterns and EMTP results suggest the need for reducing MSI morbidity and mortality through expanding emergency care training programs. Although this evidence suggests an association with improved outcomes among patients with MSI with Rwanda’s first EM residency program, further prospective evaluation of cases with MSI are needed to demonstrate reliability of these improvements over time. Moreover, similar epidemiological and training evaluation studies are needed in other African countries to effectively understand and develop scale MSI treatments.

## LIMITATIONS

Although we used formalized protocols, the design resulted in an inability to identify a proportion of cases due to incomplete medical records and some missing data among included cases, which could have biased the results. Overall, it appears that some intervention data was prioritized and thus better collected in comparison to other interventions. For example, the fact that oxygen supplementation was recorded as less used than intubation, demonstrates an inherent bias in recording interventions that are now more commonplace in the EM setting. In another example, although the GCS and vital signs in the pre-EMTP group are slightly different, it is worth noting that preliminary results show both GCS and vital signs were better recorded in the post-EMTP group vs pre-EMTP group (Table 4).

As better documentation practices were emphasized during EMTP implementation, this improvement demonstrates the inherent differences between provider training in each group, which may have led to more accurate GCS scores and vital signs in the post-EM group. The present study was performed at a single tertiary-care hospital, which may limit the generalizability of the findings to health delivery venues with less resource availability. Furthermore, due to lack of detailed information on prehospital and interfacility care provided for patients transported from various origins, controlling for prehospital interventions was not possible.

Future studies should attempt to account for such variables, especially given that a majority of patients presented from other facilities. Future studies should also attempt to differentiate patients based on varying levels of acuity, as this study’s inclusion of transfer patients likely led to a higher-acuity patient population. Additionally, general medical, technological, and other secular advances over the course of the study cannot be ignored, as healthcare does not occur in a vacuum. Many advances in Rwanda’s healthcare system have occurred in the last several years as previously noted, and the EMTP’s impact cannot be isolated due to the observational nature of this study.^16^, ^17^ However, it is worth noting from the results that changes to patient outcomes in the ED setting outperformed those same outcomes in the in-hospital setting over the same course of years, minimizing the role that technological advances played in improving outcomes. Lastly, the inclusion of patients with life-threatening injuries who also have fractures had the potential to confound results. Future research might exclude patients who require operative intervention for indications external to musculoskeletal trauma.

## CONCLUSION

This study reveals the current epidemiological foundations of MSI morbidity and mortality from a large referral center in Rwanda and the potential impacts of trained emergency physicians to properly treat those afflicted with MSI. Residency training programs such as the EMTP may be capable of helping to reduce mortality, complications, and ED length of stay among those with MSI. Such findings underscore both the efficacy and importance of investments toward educating the next generation of health professionals to treat prevalent MSI within their communities.

## Supplementary Information



## Figures and Tables

**Table 1:** Clinical characteristics of musculoskeletal injuries.

Characteristics	n (%)/median (IQR)
Fracture type	
Open	243 (35.2)
Closed	247 (35.7)
Mixed/Unknown	201 (29.1)
Anatomical regions of fracture/injuries	
Craniofacial	47 (6.8)
Thorax	27 (3.9)
Abdomen-pelvis	29 (4.2)
Spine	11 (1.6)
Upper extremity	207 (30.0)
Lower extremity	364 (52.7)
Vital signs	
Heart rate, beats per minute	88 (76–102)
Tachycardia, >100 beats per minute	70 (22.1)
Respiratory rate, breaths per minute	20 (18–20)
Tachypnea, >20 breaths per minute	15 (4.7)
Systolic blood pressure, mmHg	124 (113–137)
Hypotension, <100 mmHg	19 (6.0)
Pain scale	4 (3–6)
Neurological assessment	
Glasgow Coma Scale score	
3–8	14 (3.0)
9–12	24 (5.2)
13–15	423 (91.8)
AVPU responsiveness scale	
Alert	284 (89.6)
Verbal	8 (2.5)
Pain	2 (0.6)
Unresponsive	5 (1.6)

*IQR*, interquartile range; *mmHG*, millimeters of mercury.

**Table 2:** Care delivery for musculoskeletal injuries.

Characteristics	n (%)/median (IQR)
ED	

Emergency procedures	
Any trauma intervention	486 (76.0)
Thoracostomy	11 (1.6)
C-spine stabilization	55 (8.0)
Traction/splinting	361 (52.2)
Hemorrhage control	94 (13.6)
Wound care	151 (21.9)
IV fluid infusion	218 (34.1)
Blood products transfused	36 (16.6)
Antibiotics for all fractures	265 (84.4)
Open fractures	188 (86.6)
Tetanus shot for all fractures	169 (26.4)
Open fractures	127 (54.3)
Oxygen supplementation	64 (9.2)
Endotracheal intubation	18 (28.1)
Analgesic medication	420 (65.7)
Emergency consultations	
Total consults	579 (83.8)
Acute care surgery	119 (20.6)
Neurosurgery	58 (10.0)
Orthopedics	393 (67.9)
Other	9 (1.5)
Cases with laboratory test ordered	433 (62.8)
Cases with imaging test ordered	570 (82.5)
Admitted to the hospital	452 (73.3)
Admission ward from ED	
Surgical	125 (27.7)
ICU	6 (1.3)
Pediatrics	1 (0.2)
Neurosurgery	23 (5.1)
Orthopedics	296 (65.6)

In-hospital	

Required operative management	398 (88.1)
Laparotomy	12 (3.0)
Craniotomy	8 (2.0)
Closed reduction with external fixation	88 (22.2)
Open reduction	168 (42.3)
Wound debridement	90 (22.7)
Other	31 (7.8)
Required intensive care after admission	19 (4.2)
Hospital complications	17 (2.5)
Patient outcome	
Died in hospital	11 (2.4)
Transferred to a different health center	33 (7.3)
Unknown	3 (0.7)
Discharged	405 (89.6)

*IQR*, interquartile range; *ED*, emergency department, *IV*, intravenous; *ICU*, intensive care unit.

**Table 3:** Baseline Characteristics: Pre- and Post-EMPT.

Characteristics	Pre-EMTP	Post-EMTP	p value
Age (years)	32.0 (29.7 to 34.3)	31.0 (29.1 to 32.9)	0.5256
Female	29.0 (23.7 to 34.4)	28.2 (23.7 to 32.6)	0.8081
Lowest GCS in ED (GCS scale)	14.2 (13.9 to 14.5)	14.6 (14.4 to 14.7)	*0.0159
Open fractures	33.0 (27.4 to 38.5)	37.0 (32.2 to 41.7)	0.2868
Road traffic accidents	46.6 (40.7 to 52.5)	51.6 (46.7 to 56.6)	0.197
Vital signs			
Heart rate (beats per minute)	91.4 (88.2 to 94.5)	90.8 (88.2 to 93.3)	0.7719
Respiratory rate (breaths per minute)	20.5 (19.9 to 21.2)	19.5 (19.0 to 20.0)	*0.0115
Systolic BP (mmHG)	126.2 (123.4 to 129.1)	123.4 (120.9 to 125.9)	0.1437

p values:

* <.05

Values represent mean percentages (95% confidence interval), unless noted.

*EMTP*, emergency medicine training program; *GCS*, Glasgow Coma Scale; *BP*, blood pressure; *mmHg*, millimeters of mercury.

**Table 4:** Emergency medicine training program results.

Results & Outcomes	Pre-EMTP	Post-EMTP	p value
ED			

Deaths^	2.5 (.6 to 4.3)	0.3 (−.2 to .8)	** 0.0077
Traumatic complications^	3.6 (1.4 to 5.8)	1.0 (0.0 to 2.0)	*0.0211
Length of stay (days)^	5.3 (2.6 to 8.1)	2.5 (1.3 to 3.7)	*0.0437
Imaging requested	74.2 (69.0 to 79.4)	88.4 (85.2 to 91.5)	***<0.0001
Labs requested	55.6 (49.7 to 61.4)	69.0 (64.5 to 73.6)	***0.0003
Trauma intervention	81.8 (77.0 to 86.5)	72.4 (67.9 to 77.0)	**0.0067
IV fluid/blood given	33.1 (27.3 to 38.9)	35.4 (30.5 to 40.3)	0.5465
Tetanus antitoxin given	15.1 (10.7 to 19.5)	34.3 (29.5 to 39.2)	***<0.0001
Antibiotics given	83.1 (76.4 to 89.8)	86.9 (81.9 to 91.8)	0.3547
Analgesics given	67.3 (61.5 to 73.1)	65.1 (60.3 to 70.0)	0.5715
Injuries examined	98.9 (97.7 to 100.0)	99.7 (99.2 to 100.0)	0.1663
Consult completed	91.9 (88.4 to 95.4)	94.7 (92.2 to 97.0)	0.1853
Endotracheal intubation	34.3 (17.7 to 50.8)	20.7 (5.0 to 36.4)	0.2351
ED protocols recorded			
GCS	62.9 (57.0 to 68.7)	74.7 (70.4 to 79.1)	**0.0012
Vital signs	65.2 (59.6 to 70.9)	72.2 (67.7 to 76.6)	0.0552
Medical history	52.3 (46.4 to 58.2)	62.0 (57.2 to 66.8)	*0.0120
Physical exam	98.2 (96.6 to 99.8)	99.7 (99.2 to 100.0)	*0.0362
Patient Admitted	70.4 (64.8 to 76.0)	75.6 (71.0 to 80.1)	0.1573

In-Hospital			

Deaths	1.4 (0.0 to 2.8)	1.8 (0.5 to 3.1)	0.7331
Length of stay (days)	19.9 (17.2 to 22.7)	16.3 (13.9 to 18.7)	0.0529
Operative management	90.6 (86.3 to 94.9)	86.7 (82.6 to 90.8)	0.2082
ICU after admission	2.8 (.4 to 5.2)	4.9 (2.3 to 7.6)	0.2533

p values:

* <.05;

** <.01;

*** <.001

^ Indicates primary outcome.

Values represent mean percentages (95% confidence interval) unless otherwise noted.

*EMTP*, emergency medicine training program; *ED*, emergency department; *IV*, intravenous; *GCS*, Glasgow Coma Scale; *ICU*, intensive care unit.
