# Supplementary material for: Musculoskeletal Injuries and Outcomes Pre- and Post- Emergency Medicine Training Program
Source: West J Emerg Med. 2019 Oct 14;20(6):857–64. doi: 10.5811/westjem.2019.7.41448 (PMC6860388; doi:10.5811/westjem.2019.7.41448)
Supplement: Supplementary file 1 [file wjem-20-857-s001.docx]

SUPPLEMENTS:


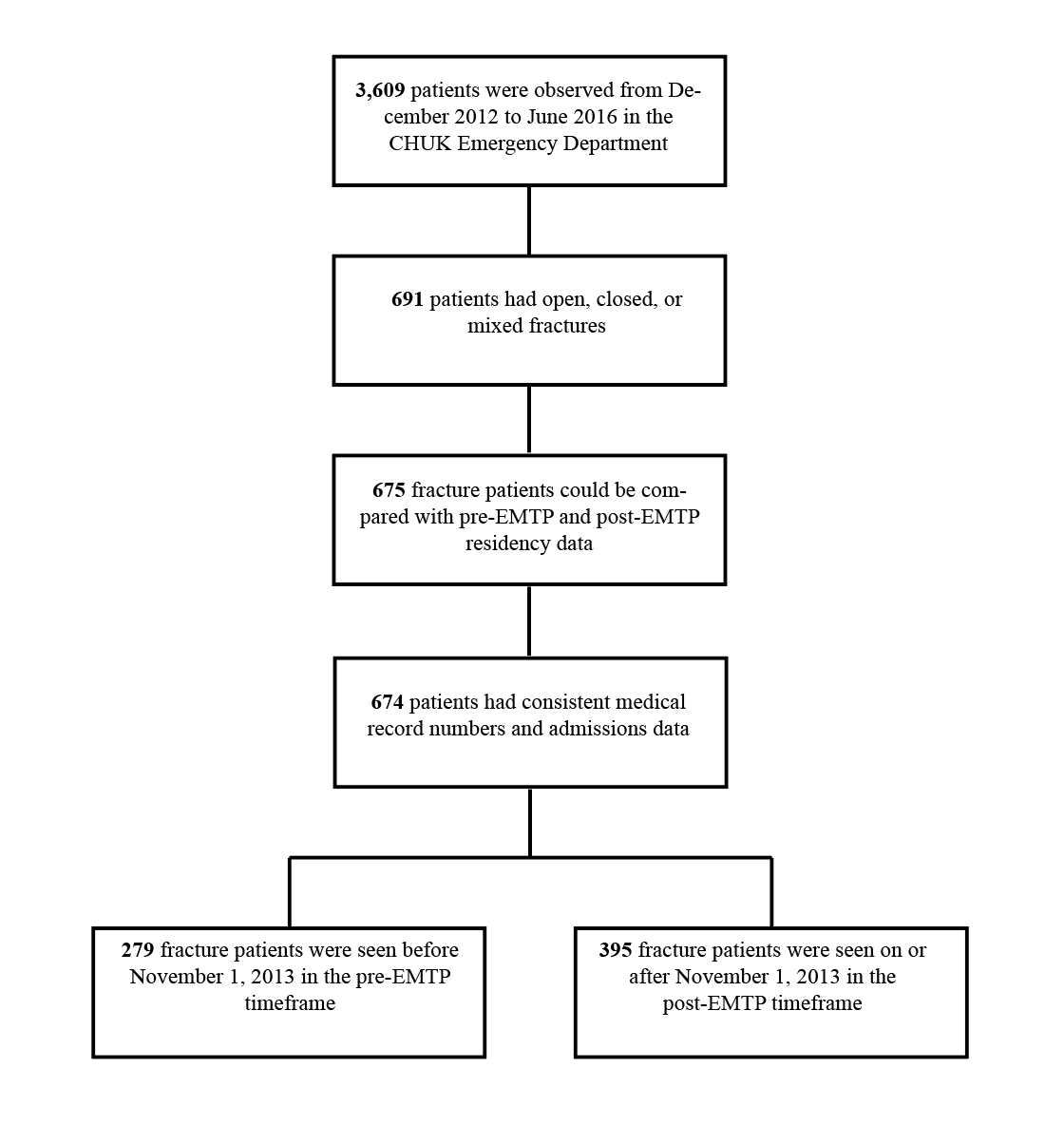


Study flow diagram. For patient selection, a total of 17 patients were excluded for incomplete or erroneous evaluation documentation dates from the ED, comprising patients without admission dates or patients with admission dates that preceded discharge dates. Of these records, 279 occurred before the start of the EMTP on November 1, 2013 while 395 occurred on or after the start of the program. Thus, patients were divided into pre-EMTP and post-EMTP groups resulting in 674 available patient records.

| MSI Cohort Characteristics | |  |
| --- | --- | --- |
| Characteristics | | n (%)/median (IQR) |
| Sex |  |  |
|  | Male | 494 (71.6) |
|  | Female | 196 (28.4) |
| Age, y | |  |
|  | Overall | 29 (18-41) |
|  | Male | 28.5 (19-38) |
|  | Female | 30 (12-49.5) |
| Age, groups | |  |
|  | 0 to 14 | 151 (21.9) |
|  | 15 to 24 | 111 (16.1) |
|  | 25 to 34 | 182 (26.3) |
|  | 35 to 44 | 99 (14.3) |
|  | 45 to 54 | 60 (8.7) |
|  | 55 to 64 | 37 (5.4) |
|  | 65+ | 51 (7.4) |
| Transported from | |  |
|  | Home | 36 (9.0) |
|  | Health Facility | 256 (64.3) |
|  | Street | 94 (23.6) |
|  | Other/unknown | 12 (3.0) |
| Type of Accident | |  |
|  | Road Traffic Accident | 336 (48.1) |
|  | Fall | 239 (34.2) |
|  | Assault (Fight, Stab, or Cut) | 42 (6.0) |
|  | Unknown | 38 (5.4) |
|  | Blunt Force | 31 (4.4) |
|  | Bombs | 4 (0.5) |
|  | Animal Encounter | 2 (0.3) |
|  | Strangulation | 1 (0.1) |
|  | Other | 5 (0.7) |
| Road Traffic Accident, Breakdown | |  |
|  | Involving vehicle | 163 (48.5) |
|  | Involving motorcycle | 145 (43.2) |
|  | Pedestrian Struck | 96 (28.6) |
|  | Pedestrian Struck (by motorcycle) | 57 (17.0) |
|  | Pedestrian Struck (by vehicle) | 39 (11.6) |
